# Postoperative Pyoderma Gangrenosum (PPG) After Appendectomy: A Case Report

**DOI:** 10.7759/cureus.42016

**Published:** 2023-07-17

**Authors:** Stanko J Baco, Jovica Mišić, Vladan Peruničić

**Affiliations:** 1 Department of General Surgery, Public Health Institution Hospital “Dr Mladen Stojanovic”, Prijedor, BIH; 2 Department of General Surgery, Saint Luke the Apostle Hospital, Doboj, BIH; 3 Department of General Surgery, General Hospital, Čačak, SRB

**Keywords:** postoperative pyoderma gangrenosum, idiopathic pyoderma gangrenosum, pathergy, corticosteroid treatment, negative-pressure wound therapy, delayed wound healing, post-appendectomy complications

## Abstract

Pyoderma gangrenosum (PG) is a rare, poorly understood, non-infectious, autoimmune phenomenon. It is an inflammatory neutrophilic dermatosis characterized by hyperactivity of the skin and the development of papules and pustules that rapidly progress to painful ulcerations with a violaceous and necrotic border. Approximately three to six cases of PG per million of the population occur per year and in the case of postoperative pyoderma gangrenosum (PPG), it is only one to three cases per million operated people.

We are presenting a 41-year-old patient with a clinical presentation of PPG, developed in the surgical site on the sixth postoperative day (POD 6) following open appendectomy for acute appendicitis. Initial treatment was for surgical site infection (SSI) with wound opening, regular dressings, and broad-spectrum antibiotics. Due to unresponsiveness to therapy and the unexpected postoperative course with the progression of skin lesions, we suspected PPG. Corticosteroid therapy was introduced in a shock dose, once daily intravenous (IV), with superb results and stopping the spread of the process after only two days.

Considering the rarity of PPG, especially when it first occurs postoperatively, we believe that the image of skin changes with superficial spreading and characteristic violaceous ulcerations can be of crucial importance for early diagnosis. A multidisciplinary approach with a mandatory examination by a dermatologist is important in order to make an early diagnosis and prevent wrong treatment, with the potential worsening of the patient's condition. Atraumatic wound care and negative pressure wound therapy are recommended. Patients at risk should perioperatively receive corticosteroids and postoperatively be closely observed for the potential development of PPG. Debridement is not recommended, and surgical treatment and further tissue trauma are undesirable and even prohibited.

## Introduction

Pyoderma gangrenosum (PG), first mentioned by Brocq in 1916 as “phagedenisme geometrique” and named in 1930 by Brunsting et al., stands for a purulent infection of the skin due to pyogenic organisms, ordinary staphylococci [1,2]. In fact, it is a misnomer since it is neither an infection nor gangrene. It is a rare non-infectious, autoimmune, inflammatory neutrophilic dermatosis. Approximately three to six cases of PG occur per year per million people. Many referral centers are seeing only one or two cases per year [3-6]. It occurs predominantly in adults (95%), mostly in the 30- to 60-year age group, and frequently in women, on any part of the body except the nipple-areolar complex [4]. In 2007, Ouzzani et al. introduced a new clinical entity, postoperative pyoderma gangrenosum (PPG), referring to skin lesion development after surgical trauma [4]. Its incidence is only one to three cases per million operated people. Only rare cases of PPG, especially as a first occurrence after appendectomy have been published in the literature [5]. An important part of the new entity is the delay between the surgical trauma and symptoms presentation, where the variation period can vary from four days to six weeks. It is characterized by the spread of changes disproportionate to the trauma, rapid progression, and lack of self-limitation [4]. There are different clinical and histological forms of this disease. It often occurs together with systemic diseases such as inflammatory bowel disease (40%) and arthritis (20%), or hematological disorders (10%) [6]. In 30% of cases, it is idiopathic [7]. The aim of this case report is to draw attention to this rare disease and help in its timely diagnosis through a presentation of the clinical development of the disease, which is, in our opinion, of paramount importance, given that there is no histopathological or laboratory specificity.

## Case presentation

A 41-year-old man presented to the emergency department complaining about abdominal pain, which lasted about one day before the presentation. On examination, we found that the abdomen was distended, tense, and tender to palpation ileocecally with peritonism. The patient denied other symptoms besides pain (such as nausea, vomiting, or fever) and had normal bowel motion. He had no allergies, had never been operated on, and had not regularly taken any medication. Laboratory test results demonstrated an elevation of inflammatory markers: white blood cell (WBC) count 22,700/μL (normal value 3,500-8,000/μL), C-reactive protein (CRP) 25.9 mg/dL (normal value < 0.3 mg/dL), red blood cell (RBC) 5.36, and Hgb 154 g/L. Deep vein thrombosis prophylaxis had been started (Clexane 0.4 ml s.c.). Perioperatively, the patient received IV 1 g of cefazolin and 0.5 g metronidazole, which is standard therapy in our institution, when we are suspecting acute appendicitis with possible perforation. He underwent emergency surgery on the same day with appendectomy and abdominal drainage. The abdomen was opened with an infraumbilical medial laparotomy. Intraoperatively, we found a perforated appendix with the local development of stercoral peritonitis and purulent collections. Despite the extensive intraoperative cleaning of the abdominal cavity and the instillation of antibiotics intraperitoneally, subfascially, and subcutaneously, the expectation of the occurrence of the wound infection was high.

Surgical site infection (SSI) developed on the third postoperative day (POD 3). The wound has been opened, a swab had been taken from the wound, and regular dressing of the wound several times during the day, without antibiotics initially followed. On POD 4, antibiotic therapy (imipenem/cilastatin 4x1 g) was started. The abdominal drain has been removed on POD 5 after a serous fluid has been obtained. Nutrition and bowel passage returned to normal.

**Figure 1:**
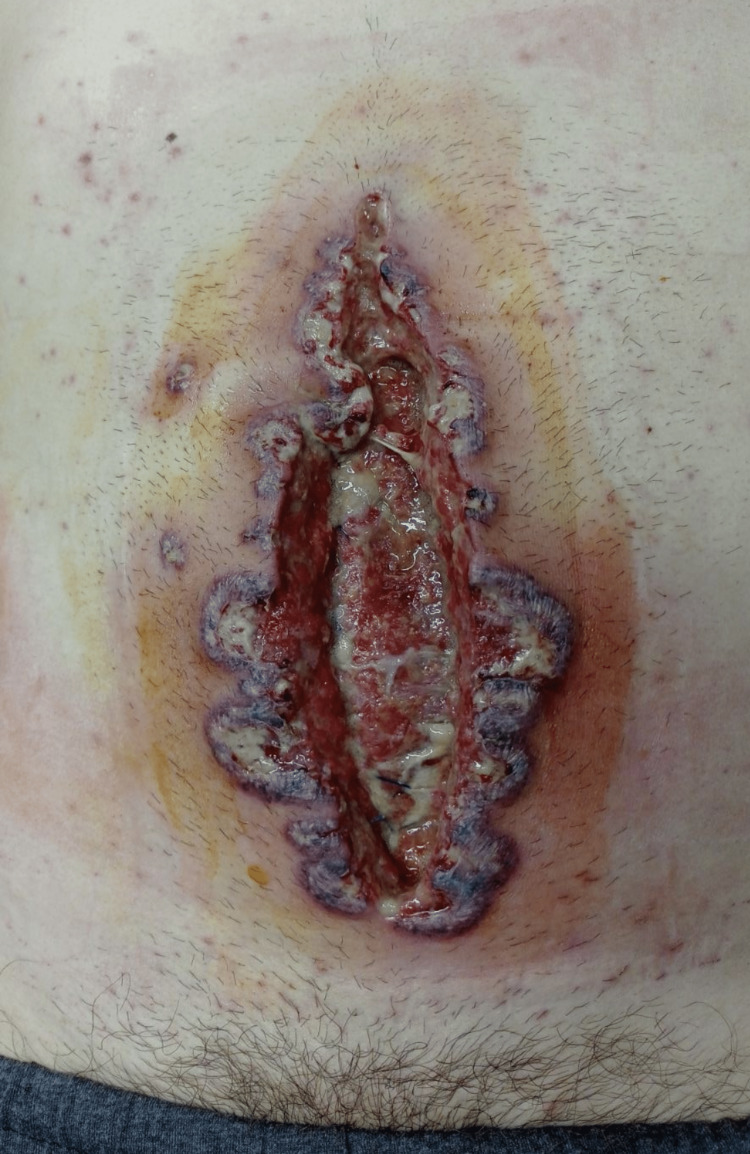
Sixth postoperative day (POD 6): infraumbilical laparotomy wound with erythematous papules and violaceous confluent wound border

**Figure 2:**
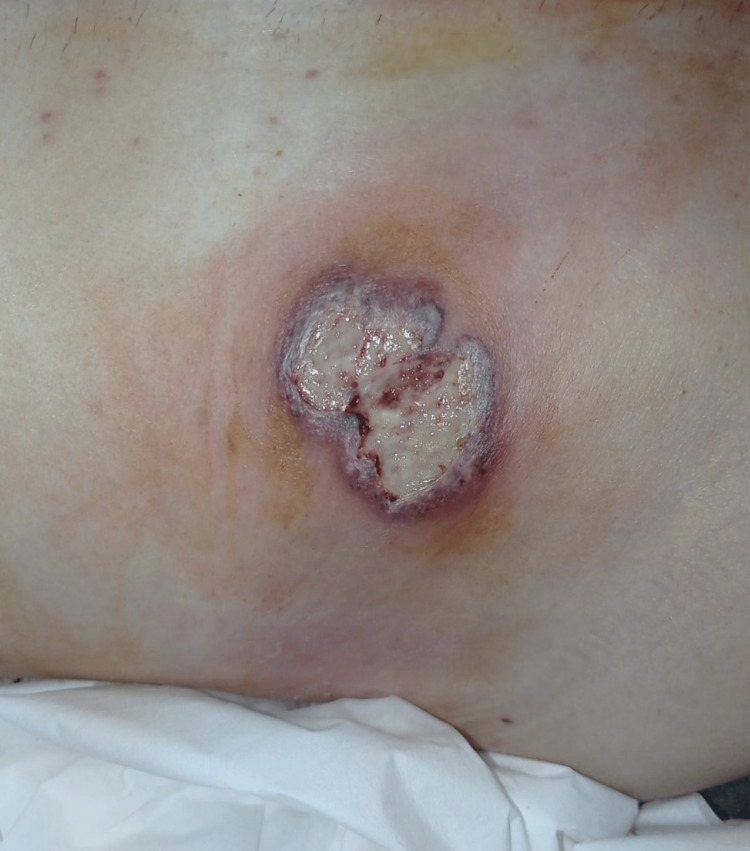
Sixth postoperative day (POD 6): exit point of the abdominal drain through the right abdominal wall with ulceration of the wound edge

The patient was hemodynamically stable all the time, without complaints, and was afebrile. On POD 6, pustules appeared on the edges of the wound as well as on the place where the abdominal drain was placed and severe pain in the wound appeared (Figures 1, 2).

**Figure 3:**
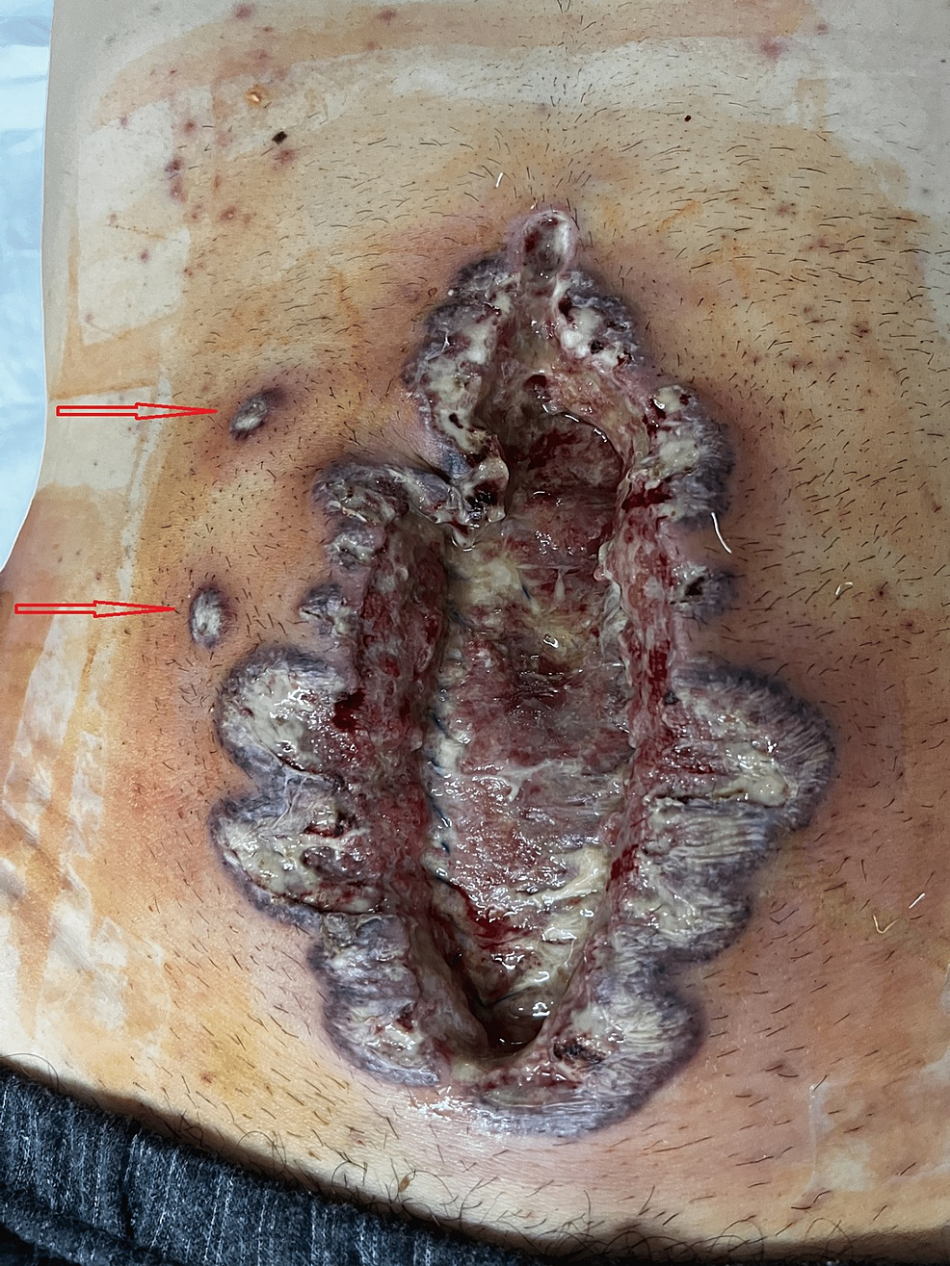
Seventh postoperative day (POD 7): medial wound with the rapid, superficial spread of the skin necrosis despite broadspectrum antibiotics Necrotic pustules simultaneously appearing at the distant site of the wound (red arrow)

**Figure 4:**
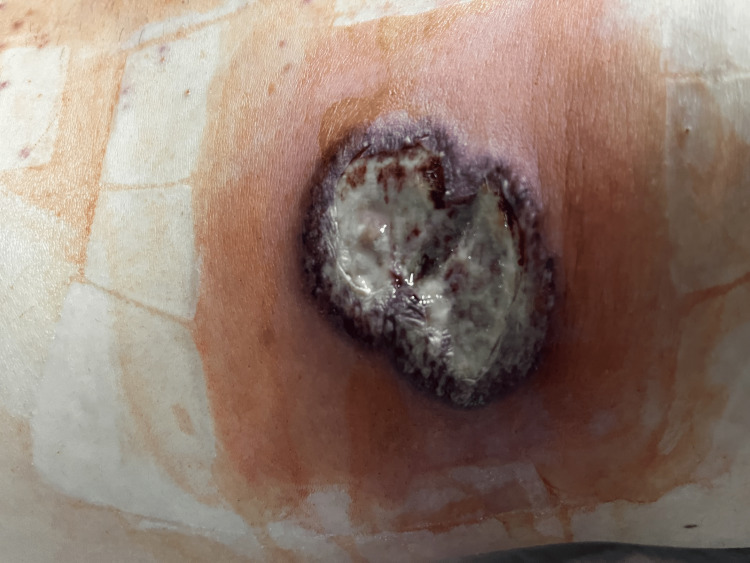
Seventh postoperative day (POD 7): exit point of the abdominal drain through the right side of the abdominal wall Violaceous and necrotic borders of the wound

Traces of Escherichia (E.) coli were isolated from the wound swab. Despite antibiotic therapy (imipenem/cilastatin 4x1 g) and regular dressing of the wound for two days, skin changes expanded rapidly. The rapid spread of pustules and formation of necrotic edges has been superficial and centrifugal. We realized that it was a pathergy phenomenon (Figures 3, 4) after having performed a pathergy test on nonglabrous skin.

**Figure 5:**
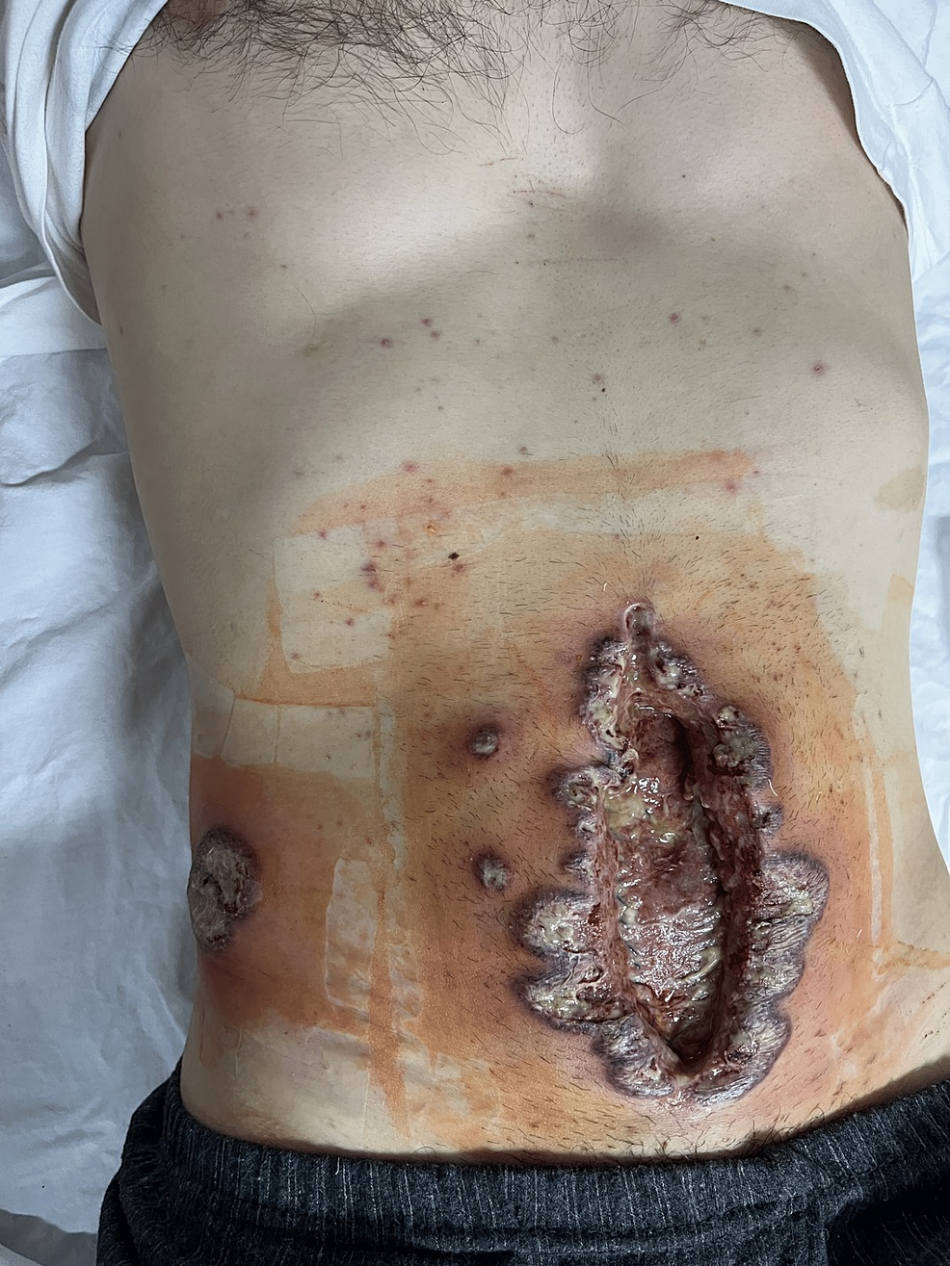
Medial laparotomy wound and exit point of the abdominal drain on the seventh postoperative day (POD 7) Rapid superficial spreading of necrotic lesions

Significant progression of the local finding happened during the weekend, so we did not have the opportunity to consult a dermatologist in the house. After an extensive consultation of the literature, we suspected PG and consulted a dermatologist by video call, presenting the findings and clinical course to him and showing the pictures of the changes. A deep elliptical skin biopsy, including skin and subcutaneous tissue, had been obtained under local anesthesia and sent for histopathological examination (Figure 5).

SSI was ruled out clinically, when after opening the wound with evacuation of pus and regular dressings, the wound soon became clean, with clean walls and the base of the wound. Despite that, a superficial, rapid progression of the destruction of the epidermis with a clear demarcation of skin changes developed. Additional factors to exclude SSI were an almost complete absence of bacteria in the wound swab and no response and stopping to the spread of skin changes despite the administration of broad-spectrum antibiotics.

After receiving an affirmative opinion from the dermatologist, we started systemic immunosuppressive therapy. Corticosteroid therapy was introduced in a shock dose, 100 mg of methylprednisolone IV, once daily. After only two days, the progression of skin changes stopped. We received the pathohistological findings of the biopsy of the wound after a few weeks, which showed the presence of neutrophils and granulocytes. The histological picture could support the diagnosis of pyoderma gangrenosum.

Due to relatives' concerns about the patient's condition, transfer to a tertiary-level hospital followed after two days. Wound debridement was done, corticosteroids were excluded from therapy, and negative pressure wound therapy and vacuum-assisted closure (VAC) were administered. The further course of the disease was uneventful, the wound reduced regularly in size and after eight days, the VAC system was removed and the wound sutured. The wound healed completely after one month. Further diagnostics and follow-up of the patient, in terms of proving the existence of concomitant, autoimmune, hematological, or malignant diseases, was not possible because we lost the patient during the follow-up (the patient lived in another state entity, where he continued his life).

## Discussion

Pyoderma gangrenosum is a rare, poorly understood immunologic disorder. It is an inflammatory neutrophilic dermatosis, characterized by painful and erythematous papules, pustules, or vesicles that rapidly progress to ulcerations with a violaceous and necrotic border [8]. It can affect any anatomical location with the exception of the nipple-areolar complex [4]. The pathergy phenomenon is characteristic of PG and PPG and should raise suspicion. It refers to skin rapidly developing lesions after minor trauma. The underlying principle is the hyperactivity of the skin, with uncontrolled neutrophilic inflammation as the cornerstone, and as a result of the excessive release of Interleukin-8 (IL-8) mediators in a genetically predisposition individual [9]. That leads to the rapid progression and development of skin necrosis, single or multiple, of different sizes without self-limitation, which is an important factor [4]. Clinically, the patient complains of severe pain in the incision area. PPG occurred most commonly after breast, cardiothoracic, abdominal, and obstetric (13%) surgeries [10].

The most common associated disease with which PG presents is an inflammatory bowel disease (40%). MD Levitt et al. had shown in their paper that PG occurs in both ulcerative colitis and Crohn's disease and that the healing process after intestinal resection is unpredictable [11].

Specific findings in the skin of the wound, an almost sterile finding on the wound swab, being unresponsive to antibiotics, and the rapid progression of skin changes should raise suspicion. The diagnosis is established by a combination of rational thinking, excluding similar diseases and fulfilling some of the known criteria (one major and at least two minor criteria) established by the Delphi Consensus of International Experts [12]. One major criterion is the biopsy with the neutrophilic infiltrate. Minor criteria are (1) exclusion of infection, (2) pathergy phenomenon, (3) associated autoimmune disease, (4) peripheral erythema and tenderness at ulceration, (5) multiple ulcerations (which are not directly related to the incision site), (6) papules or pustules ulcerating within four days of appearing, (7) cribriform or “wrinkled paper” scar(s) at healed ulcer sites, and (8) good response to systemic immunosuppression therapy with decreasing ulcer size [12]. In our presented case report, one major and five out of eight minor criteria have been fulfilled, yielding a sensitivity and specificity of 86% and 90%, respectively [12]. In this case report, we are focused on the clinical presentation and differential diagnosis. SSI is a differential diagnostic consideration. Especially in our case, where after the perforation of the appendix, despite the extensive cleaning of the abdominal cavity and the instillation of antibiotics intraperitoneally, subfascially, and subcutaneously, the occurrence of wound infection was very high. The development of wound infection that can be expected after stercoral peritonitis may initially mimic PG. Drug-induced pyoderma gangrenosum, which should also be considered, was unlikely since the patient did not regress even after stopping all drugs. Considering how rare PG is, especially PPG as the first episode of the disease, when skin changes are the first sign of the disease, without the existence or knowledge of an associated autoimmune disease, we believe that the picture of skin changes is of crucial importance for quick diagnostics. All the more so because in only 25-50% of cases a concomitant autoimmune disease is found while the histopathological picture is nonspecific. The principle guiding wound care in a patient with PG should be the avoidance of undue trauma or irritation of the wound surface and the prevention of secondary infection. If the surgery must be performed, it should be when the lesions are clinically inactive and debridement of the wound should be done only in the setting of extensive necrosis or uncontrollable superinfection [8,10,13].

Negative pressure wound therapy (NPWT) is considered a safe option for adjuvant treatment of wounds caused by PG, especially when combined with systemic immunosuppression. It plays an immense role in successful treatment and accelerating wound closure, and based on that evidence, NPWT was applied [14]. 

## Conclusions

Considering how rare PSPG is, especially when it first occurs postoperatively, we believe that the image of skin changes with superficial spreading and characteristic merging ulcerations can be of crucial importance for early diagnosis. A multidisciplinary approach with a mandatory examination by a dermatologist is important to make an early diagnosis and prevent wrong treatment with potential worsening of the patient's condition. Suspicion should be aroused especially in situations where there is a discrepancy between the clinical picture and the expected course of the disease or pathergy phenomenon. An unexpected postoperative course with an unusual, uncharacteristic finding of centrifugal progression of skin lesions should raise the suspicion of pyoderma gangrenosum. Atraumatic wound care and negative pressure wound therapy are recommended and can mitigate unnecessary morbidity. Patients at risk should perioperatively receive corticosteroids and postoperatively be closely observed for the potential development of PPG. Debridement is not recommended, and surgical treatment and further tissue trauma are undesirable and even prohibited.
